# Supplementary material for: A fault-intrusion-tolerant system and deadline-aware algorithm for scheduling scientific workflow in the cloud
Source: PeerJ Comput Sci. 2021 Nov 2;7:e747. doi: 10.7717/peerj-cs.747 (PMC8576554; doi:10.7717/peerj-cs.747)
Supplement: Supplemental Information 6 [file peerj-cs-07-747-s006.zip › Myproject1.1/docs/org/workflowsim/scheduler/class-use/SchedulerInterface.html]

Uses of Interface org.workflowsim.scheduler.SchedulerInterface


JavaScript is disabled on your browser.


- Overview
- Package
- Class
- Use
- Tree
- Deprecated
- Index
- Help

- Prev
- Next

- Frames
- No Frames

- All Classes

## Uses of Interface org.workflowsim.scheduler.SchedulerInterface

- Packages that use SchedulerInterface

  | Package | Description |
  | --- | --- |
  |  |  |
  | --- | --- |
  | org.workflowsim.scheduler |  |
- - ### Uses of SchedulerInterface in org.workflowsim.scheduler

    Classes in org.workflowsim.scheduler that implement SchedulerInterface

    | Modifier and Type | Class and Description |
    | --- | --- |
    |  |  |
    | --- | --- |
    | `class` | `BaseScheduler` The base scheduler has implemented the basic features. |
    | `class` | `DataAwareScheduler` Data aware algorithm. |
    | `class` | `FCFSScheduler` The FCFS algorithm. |
    | `class` | `MaxMinScheduler` MaxMin algorithm. |
    | `class` | `MCTScheduler` MCT algorithm |
    | `class` | `MinMinScheduler` MinMin algorithm. |
    | `class` | `StaticScheduler` Static algorithm. |

- Overview
- Package
- Class
- Use
- Tree
- Deprecated
- Index
- Help

- Prev
- Next

- Frames
- No Frames

- All Classes
